# Author Correction: PLMSearch: Protein language model powers accurate and fast sequence search for remote homology

**DOI:** 10.1038/s41467-024-52177-w

**Published:** 2024-09-05

**Authors:** Wei Liu, Ziye Wang, Ronghui You, Chenghan Xie, Hong Wei, Yi Xiong, Jianyi Yang, Shanfeng Zhu

**Affiliations:** 1 Institute of Science and Technology for Brain-Inspired Intelligence and MOE Frontiers Center for Brain Science, Fudan University, 200433 Shanghai, China; 2 School of Mathematical Sciences, Fudan University, 200433 Shanghai, China; 3 School of Mathematical Sciences, Nankai University, 300071 Tianjin, China; 4 Department of Bioinformatics and Biostatistics, Shanghai Jiao Tong University, 200240 Shanghai, China; 5 Ministry of Education Frontiers Science Center for Nonlinear Expectations, Research Center for Mathematics and Interdisciplinary Science, Shandong University, 266237 Qingdao, China; 6 Shanghai Qi Zhi Institute, Shanghai, China; 7 Key Laboratory of Computational Neuroscience and Brain-Inspired Intelligence (Fudan University), Ministry of Education, Shanghai, China; 8 Shanghai Key Lab of Intelligent Information Processing and Shanghai Institute of Artificial Intelligence Algorithm, Fudan University, Shanghai, China; 9 Zhangjiang Fudan International Innovation Center, Shanghai, China

**Keywords:** Bioinformatics, Software, Protein sequencing, Computational models, Protein sequence analyses

Correction to: *Nature Communications* 10.1038/s41467-024-46808-5, published online 30 March 2024

The original version of this article omitted an attribution to previous work by Kaminski et al., 2023. This has been added as reference (Ref. 55) in the Methods: “Specifically, inspired by pLM-BLAST [48, 55], PLMAlign also uses per-residue embeddings of the query-target protein pair to calculate the substitution matrix.” and “On the other hand, compared with pLM-BLAST, which uses cosine similarity, PLMAlign employs the dot product similarity and linear gap penalty. This enables PLMAlign to better align remote homology pairs while reducing the algorithm’s complexity to O(mn) to ensure high efficiency. The other differences between the SW/NW algorithm, pLM-BLAST, and PLMAlign are discussed in further detail in Supplementary Table 14.”

In the Code Availability, a declaration of the modification of the pLM-BLAST code has been added: “whose pipeline is modified from pLM-BLAST [48, 55]. pLM-BLAST is available from https://github.com/labstructbioinf/pLM-BLAST under an MIT License that allows the use, copying, modification, merging and publishing of the software, with copyright notice and permission notice included in all copies or substantial portions of the software.”

These have been corrected in both the PDF and HTML versions of the Article.

