# Supplementary material for: PLMSearch: Protein language model powers accurate and fast sequence search for remote homology
Source: Nat Commun. 2024 Mar 30;15:2775. doi: 10.1038/s41467-024-46808-5 (PMC10981738; doi:10.1038/s41467-024-46808-5)
Supplement: Supplementary file 1 — Supplementary Information [file 41467_2024_46808_MOESM1_ESM.pdf]

PLMSearch: Protein language model powers accurate and fast sequence search for remote homology

Wei Liu[1], Ziye Wang[1], Ronghui You[1], Chenghan Xie[2], Hong Wei[3], Yi Xiong[4], Jianyi Yang[5*] and Shanfeng Zhu[1,6,7,8,9*]

[1]Institute of Science and Technology for Brain-Inspired Intelligence and MOE Frontiers Center for Brain Science, Fudan University, Shanghai, 200433, China.
[2]School of Mathematical Sciences, Fudan University, Shanghai, 200433, China.
[3]School of Mathematical Sciences, Nankai University, Tianjin, 300071, China.
[4]Department of Bioinformatics and Biostatistics, Shanghai Jiao Tong University, Shanghai, 200240, China.
[5]Ministry of Education Frontiers Science Center for Nonlinear Expectations, Research Center for Mathematics and Interdisciplinary Science, Shandong University, Qingdao, 266237, China.
[6]Shanghai Qi Zhi Institute, Shanghai, China.
[7]Key Laboratory of Computational Neuroscience and Brain-Inspired Intelligence (Fudan University), Ministry of Education, Shanghai, China.
[8]Shanghai Key Lab of Intelligent Information Processing and Shanghai Institute of Artificial Intelligence Algorithm, Fudan University, Shanghai, China.
[9]Zhangjiang Fudan International Innovation Center, Shanghai, China.

*Corresponding author(s). E-mail(s): yangjy@sdu.edu.cn; zhusf@fudan.edu.cn;

1 Supplementary Figures and Tables

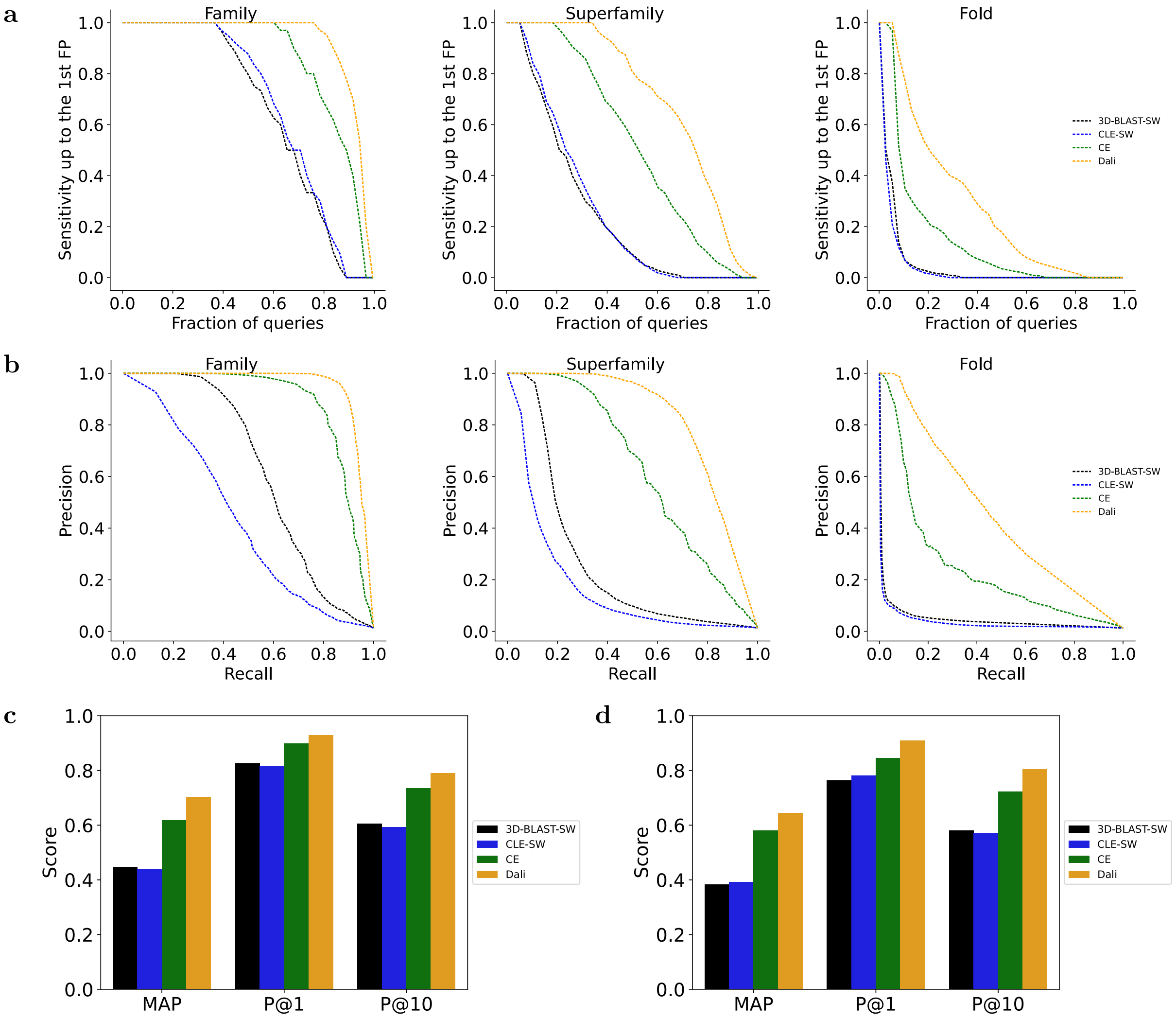

Supplementary Fig. 1 Evaluation of other baselines. a-c, The all-versus-all search test on SCOPe40-test. **d,** Evaluation on new proteins (see "New protein search test" Section). Supplementary Table 2 and Supplementary Table 4 record the specific values of each metric. Source data are provided as a Source Data file.

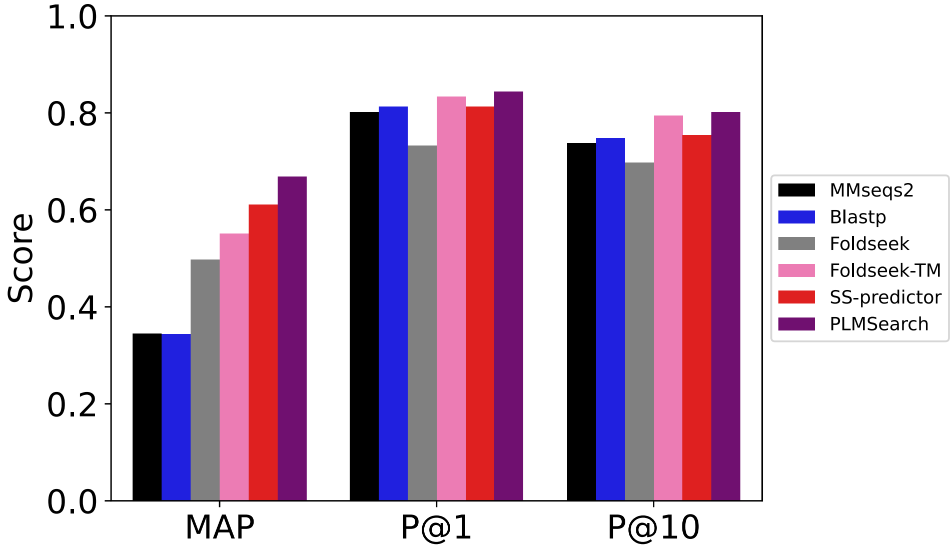

Supplementary Fig. 2 MAP, P@1, and P@10 on the search test with Swiss-Prot as the target dataset. Supplementary Table 1 records the specific values of each metric. Source data are provided as a Source Data file.

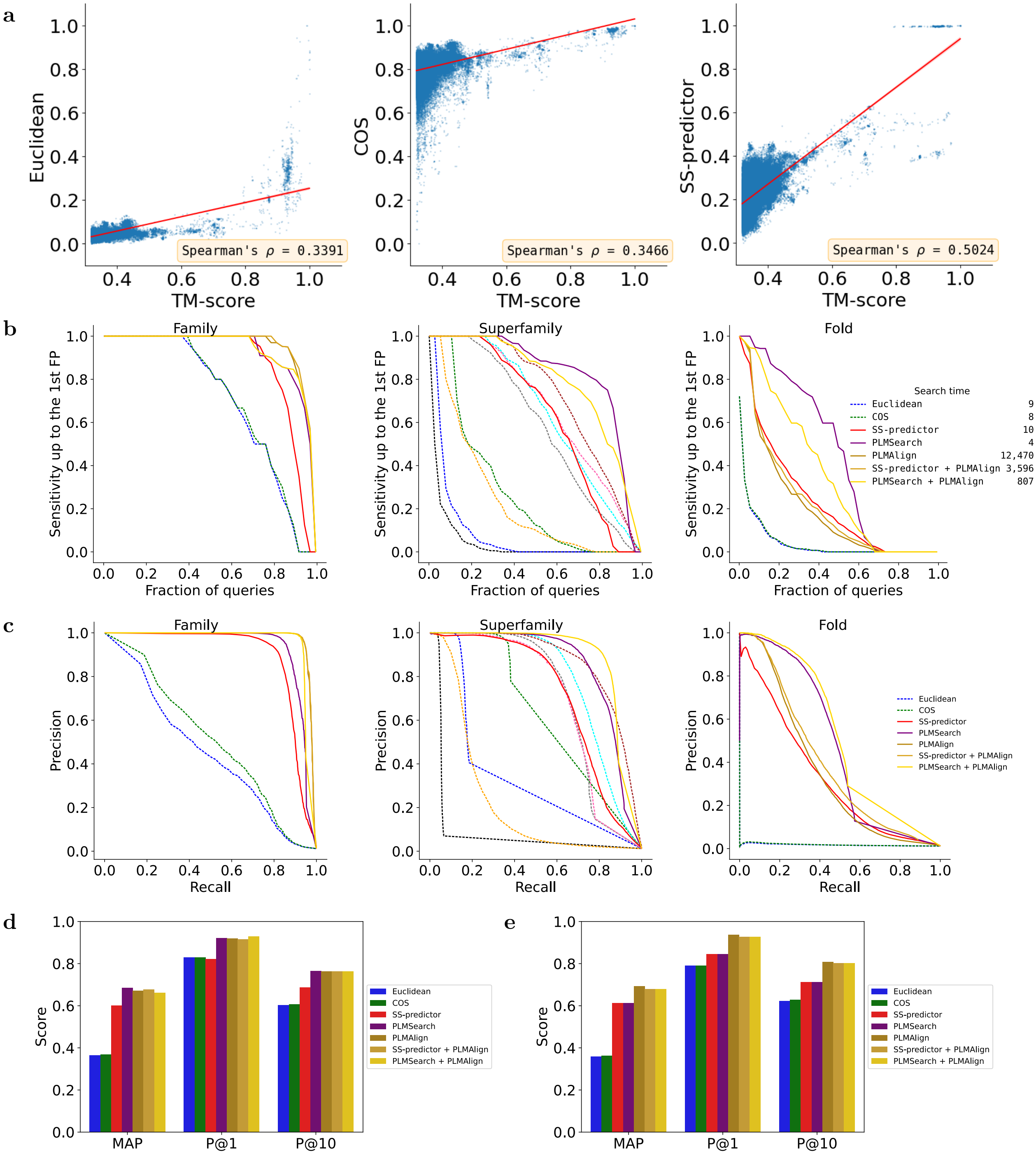

Supplementary Fig. 3 Ablation experiments: PfamClan, SS-predictor, and PLMAlign make PLMSearch more robust. a, Two-dimensional scatter plot of the predicted similarity and TM-score. From left to right are Euclidean, COS, and SS-predictor. We selected 100,000 protein pairs with the highest TM-scores from the search results of five queries (with Swiss-Prot as the target dataset, 100,000 among a total of 2,150,700 query-target pairs) and used Euclidean, COS, and SS-predictor as the predicted similarity. We normalized the predicted similarity to 0-1 as the y-axis and their TM-scores (between 0-1) as the x-axis, thereby plotting the 100,000 protein pairs as points on a 2D plane. SS-predictor obtained the highest correlation coefficient with TM-score. **b-e,** Ablation experiments, with the same metrics used in Fig. 2 in the main text. Supplementary Table 2 and Supplementary Table 4 record the specific values of each metric. Source data are provided as a Source Data file.

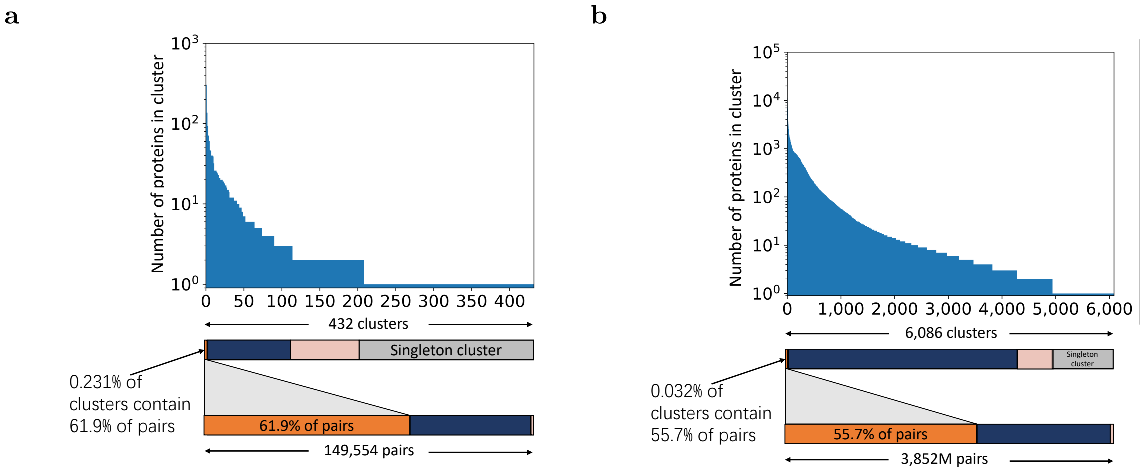

Supplementary Fig. 4 Clustering results based on Pfam clan on SCOPe40-test and Swiss-Prot. a, SCOPe40-test. **b,** Swiss-Prot. Proteins belonging to the same Pfam clan are clustered. The clustering results show a significant long-tailed distribution. After pre-filtering with PfamClan, more than 50% of the pre-filtered protein pairs (orange rectangles in the figure) are from the largest 1-2 clusters (big clusters), which only accounts for a very small part of the entire clusters (SCOPe40-test: 0.231%; Swiss-Prot: 0.032%). Therefore, big clusters will result in a significant number of irrelevant protein pairs in the pre-filtering results, reducing accuracy, and must be further sorted and filtered based on similarity, which is what SS-predictor does. See Supplementary Table 8 for specific statistical data. Source data are provided as a Source Data file.

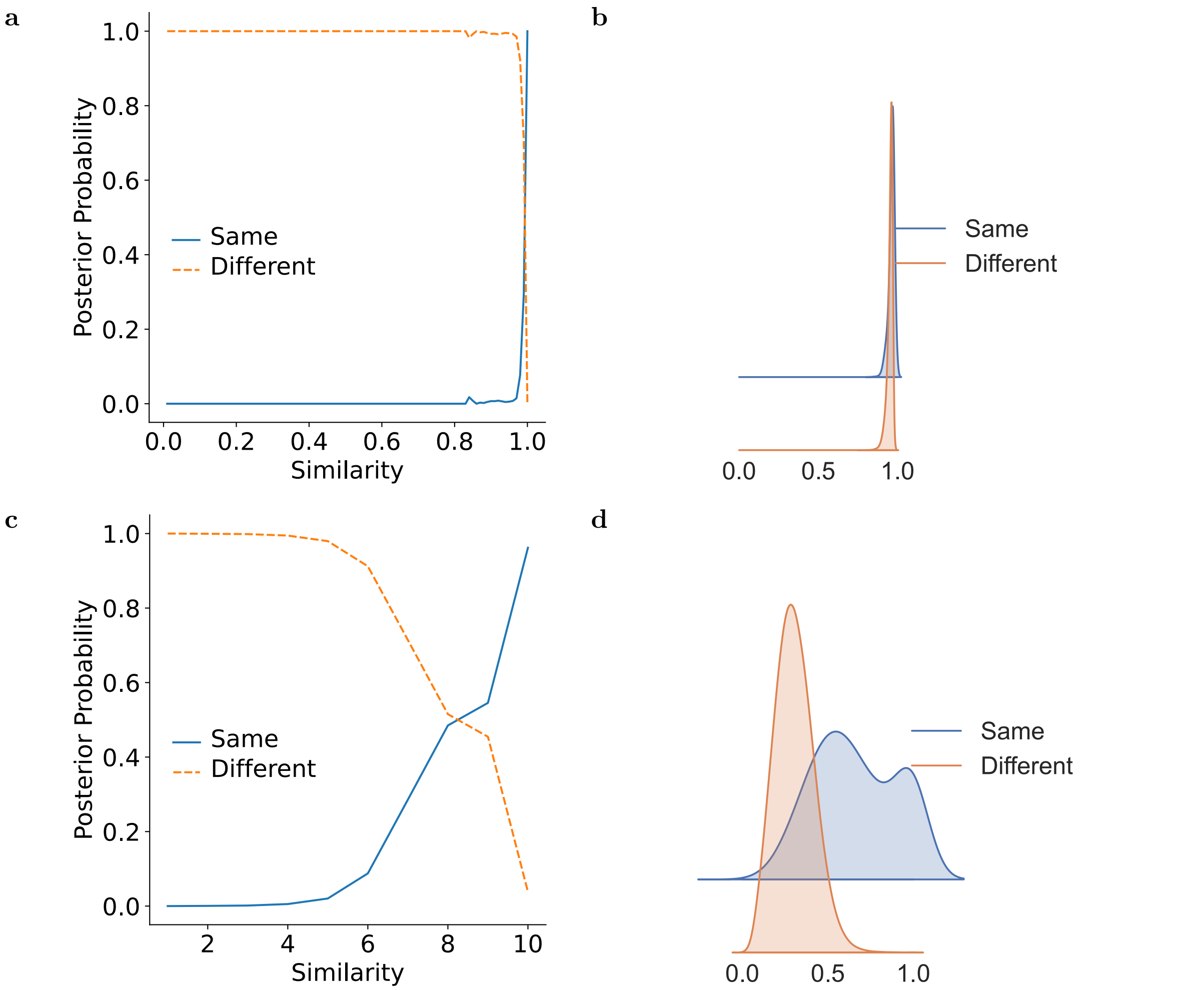

Supplementary Fig. 5 Reference value of COS similarity and PLMAlign score. **a-b**, COS similarity. **c-d**, PLMAlign score. **a, c** show the posterior probability of proteins with a given similarity being in the same fold or different folds in SCOPe40-train. **b, d** show the similarity distribution of the same fold and different folds protein pairs using kernel density estimation (smoothed histogram using a Gaussian kernel with the width automatically determined). The posterior probability corresponding to the similarity is shown in Supplementary Table 12. See "Reference similarity" Supplement Section for more details.

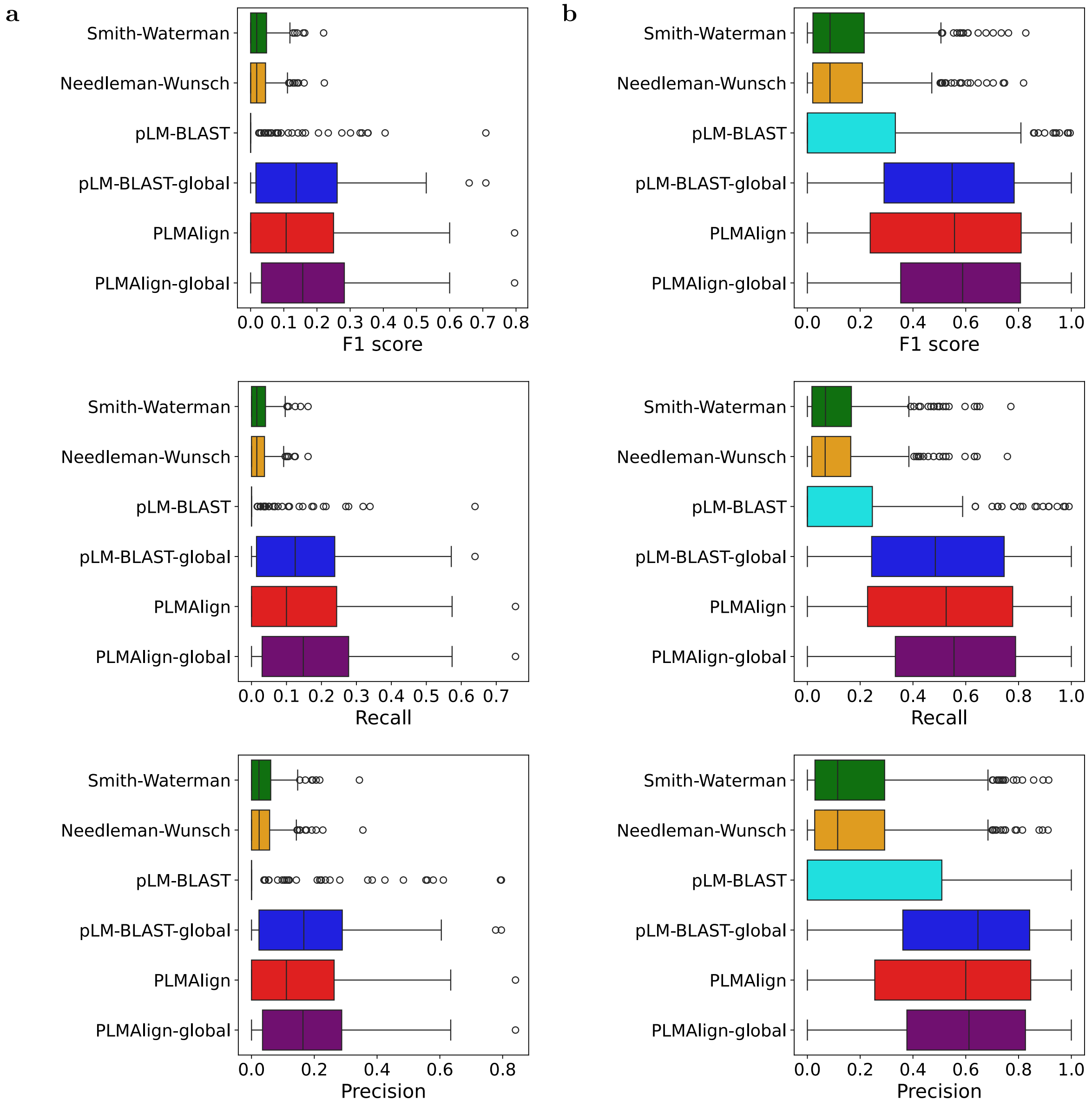

Supplementary Fig. 6 Evaluation on remote homology alignment. a, Malisam. **b,** Malidup. Figures here use the embedding generated by ProtT5-XL-UniRef50 as input. Supplementary Table 15 records the specific values of each metric. Source data are provided as a Source Data file.

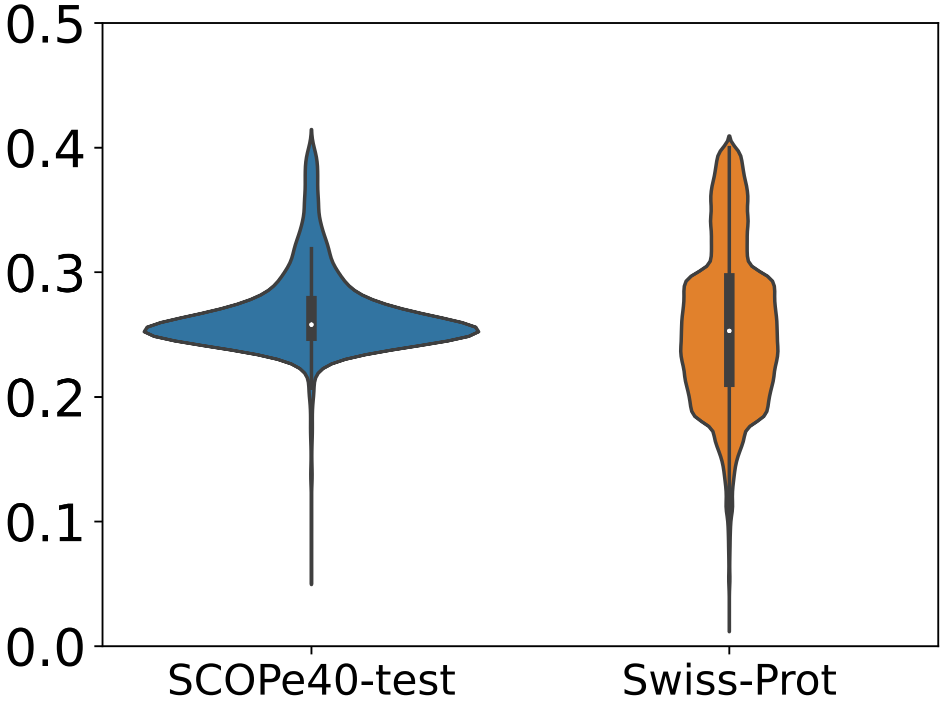

Supplementary Fig. 7 The data distribution of max sequence identity of each protein in the test dataset against the training dataset (Supplementary Table 16). The majority of the maximum sequence identity is between 0.2 and 0.3. The sequence identity difference between their data is significantly bigger than that of pure random division, especially for the SCOPe40-test, which is the major test data, since the domains in SCOPe40-test belong to different folds with all domains in SCOPe40-train.

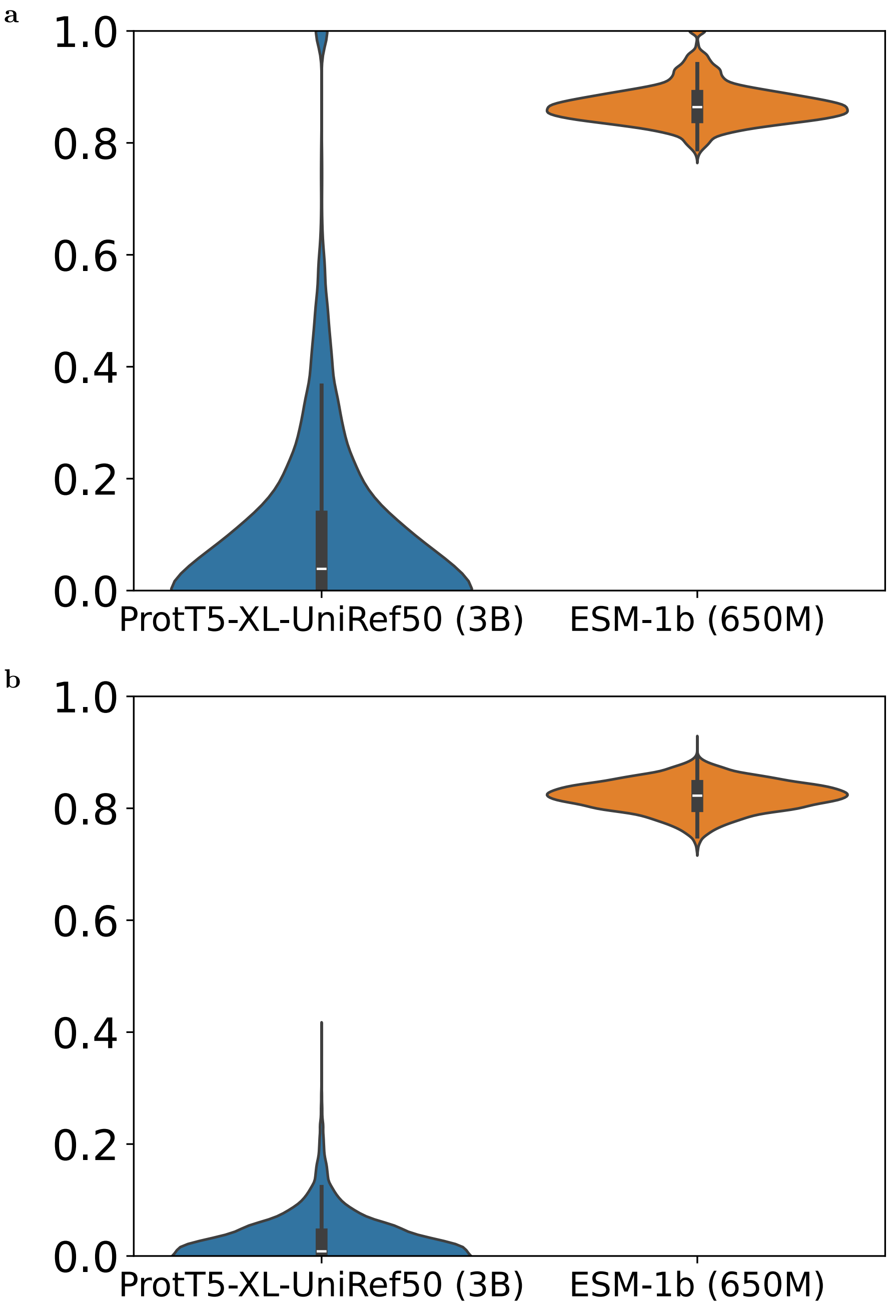

Supplementary Fig. 8 Comparison of ESM-1b and ProtT5-XL-UniRef50. The cos distance between the per-residue embeddings of two proteins. **a,** Self-alignment ($n*n$). **b,** Alignment with another protein ($n*m$). The COS distance between embeddings generated by ProtT5-XL-UniRef50 has better discrimination, both in self-alignment and alignment with another protein.

	MAP	P@K	
Methods	MAP	P@1	P@10
Baselines			
MMseqs2	0.345	0.802	0.737
Blastp	0.343	0.812	0.748
Foldseek	0.497	0.732	0.697
Foldseek-TM	0.551	0.833	0.794
Our methods			
SS-predictor	0.610	0.812	0.754
PLMSearch	**0.668**	**0.843**	**0.801**

Supplementary Table 1 Search test with Swiss-Prot as the target dataset. TPs are protein pairs with TM-scores higher than 0.5. The definition of MAP and P@K is detailed in "Metrics" Section. The highest value achieved for each metric is highlighted in bold.

	AUROC			AUPR			MAP	P@K		Time
Methods	Fam	Sfam	Fold	Fam	Sfam	Fold	MAP	P@1	P@10	Seconds
Sequence search										
MMseqs2	0.318	0.050	0.002	0.430	0.091	0.014	0.147	0.668	0.260	**2 s**
Blastp	0.527	0.161	0.004	0.717	0.342	0.029	0.183	0.717	0.354	10 s
HHblits	0.920	0.363	0.064	0.969	0.623	0.256	0.320	0.858	0.577	10,998 s
EAT	0.648	0.230	0.025	0.646	0.225	0.020	0.350	0.813	0.575	27 s
pLM-BLAST	0.940	0.642	0.176	0.973	0.779	0.305	0.659	0.921	0.760	18,812 s
Structure search — structural alphabet										
3D-BLAST-SW	0.653	0.255	0.045	0.621	0.264	0.047	0.446	0.825	0.604	-
CLE-SW	0.672	0.265	0.033	0.432	0.171	0.035	0.440	0.814	0.592	-
Foldseek	0.883	0.584	0.214	0.921	0.703	0.320	0.598	0.908	0.751	12 s
Foldseek-TM	0.898	0.664	0.296	0.906	0.695	0.337	0.626	0.905	0.756	173 s
Structure search — structural alignment										
CE	0.847	0.527	0.148	0.882	0.627	0.245	0.618	0.897	0.734	-
Dali	0.923	0.702	0.281	0.948	0.814	0.454	0.702	0.927	0.790	-
TM-align	0.935	0.721	0.346	0.971	0.866	**0.569**	**0.781**	**0.941**	**0.806**	11,303 s
Our methods										
Euclidean	0.699	0.309	0.039	0.456	0.107	0.016	0.364	0.829	0.603	9 s
COS	0.705	0.316	0.040	0.514	0.130	0.017	0.367	0.830	0.606	8 s
SS-predictor	0.869	0.623	0.225	0.891	0.713	0.324	0.601	0.821	0.686	10 s
PLMSearch	0.928	**0.826**	**0.438**	0.931	0.849	0.473	0.685	0.922	0.765	4 s
PLMAlign	0.946	0.652	0.196	0.974	0.807	0.354	0.670	0.919	0.763	12,470 s
SS-predictor + PLMAlign	**0.949**	0.665	0.211	**0.975**	0.822	0.391	0.677	0.915	0.763	3,596 s
PLMSearch + PLMAlign	0.933	0.787	0.342	0.956	**0.887**	0.521	0.660	0.928	0.763	807 s

Supplementary Table 2 All-versus-all search test on the SCOPe40-test dataset. The definition of AUROC, AUPR, MAP, and P@K is detailed in "Metrics" Section. The highest value achieved is highlighted in bold. Due to the width limit, Family and Superfamily are abbreviated as Fam and Sfam in the table, respectively. The total search time spent for the all-versus-all search test is recorded.

Methods	Family	Superfamily	Fold	Total
Sequence search				
MMseqs2	2.20	0.51	0.02	2.74
Blastp	4.39	1.21	0.05	5.65
HHblits	9.10	11.25	3.92	24.29
EAT	6.17	6.12	1.65	13.96
pLM-BLAST	8.96	30.57	13.95	53.50
Structure search — structural alphabet				
3D-BLAST-SW	6.95	8.92	1.44	17.32
CLE-SW	6.97	9.65	1.10	17.74
Foldseek	8.57	28.33	14.19	51.10
Foldseek-TM	8.68	34.62	20.79	64.11
Structure search — structural alignment				
CE	8.70	23.82	8.72	41.25
Dali	9.23	33.62	16.74	59.60
TM-align	9.29	38.57	24.83	72.70
Our methods				
Euclidean	6.88	8.52	1.70	17.12
COS	6.94	8.79	1.77	17.52
SS-predictor	8.78	34.88	20.62	64.29
PLMSearch	**9.32**	**47.44**	**48.01**	**104.78**
PLMAlign	9.04	32.40	16.68	58.12
SS-predictor + PLMAlign	9.10	33.60	18.27	60.97
PLMSearch + PLMAlign	9.11	41.50	32.07	82.69

Supplementary Table 3 The average number of family TPs, superfamily TPs, fold TPs, and total TPs up to the first FP on the SCOPe40-test search test. The average number of the total TPs also means the average rank of the first FP. The highest value achieved is highlighted in bold.

	MAP	P@K		Time
Methods	MAP	P@1	P@10	Seconds
Sequence search				
MMseqs2	0.107	0.518	0.149	**0.1 s**
Blastp	0.132	0.590	0.270	0.5 s
HHblits	0.279	0.872	0.581	548.1 s
EAT	0.315	0.790	0.576	1.3 s
pLM-BLAST	0.682	0.936	0.805	937.6 s
Structure search — structural alphabet				
3D-BLAST-SW	0.383	0.763	0.580	-
CLE-SW	0.392	0.781	0.571	-
Foldseek	0.521	0.863	0.730	0.6 s
Foldseek-TM	0.560	0.881	0.740	8.6 s
Structure search — structural alignment				
CE	0.580	0.845	0.722	-
Dali	0.643	0.909	0.804	-
TM-align	**0.776**	**0.945**	**0.826**	563.3 s
Our methods				
Euclidean	0.358	0.790	0.621	0.4 s
COS	0.363	0.790	0.628	0.4 s
SS-predictor	0.612	0.845	0.712	0.5 s
PLMSearch	0.612	0.845	0.712	0.5 s
PLMAlign	0.692	0.936	0.807	621.5 s
SS-predictor + PLMAlign	0.679	0.927	0.801	179.2 s
PLMSearch + PLMAlign	0.679	0.927	0.801	179.2 s

Supplementary Table 4 Evaluation on new proteins. See “New protein search test” Section. The definition of MAP, P@K is detailed in “Metrics” Section. The highest value achieved is highlighted in bold. The total search time spent for the search test is recorded.

Query num	1	10	100
Swiss-Prot (568K proteins)			
SS-predictor	0.2 min	0.5 min	10.3 min
PLMSearch	0.2 min	1.1 min	15.6 min
UniRef50 (53.6M proteins)			
SS-predictor	1.6 min	6.3 min	60.2 min
PLMSearch	2.3 min	12.1 min	114.6 min

Supplementary Table 5 Total running time of the web server. The environment of the web server is CPU ONLY, with 64 * Intel(R) Xeon(R) CPU E5-2682 v4 @ 2.50 GHz and 512 GB RAM. The time required to search 1, 10, and 100 query proteins with Swiss-Prot (568K proteins, the original dataset without filtering) and UniRef50 (53.6M proteins) as the target dataset were counted respectively.

Methods	Search				5. Alignment	Total
	1. Query embedding	2. Query pfam	3. Pfamclan	4. SS-predictor		
Swiss-Prot (568K proteins)						
SS-predictor	65	0	0	41	513	619
PLMSearch	65	35	29	28	779	936
UniRef50 (53.6M proteins)						
SS-predictor	62	0	0	3,006	548	3,616
PLMSearch	62	38	3,324	2,893	563	6,880

Supplementary Table 6 Running time (Seconds) of the web server at each step. Search 100 query proteins with Swiss-Port (568K proteins) and UniRef50 (53.6M proteins) as the target dataset.

	All pairs		Easy pairs		Remote homology pairs	
Methods	Recall	Miss	Recall	Miss	Recall	Miss
MMseqs2	0.450	0.549	**1.000**	**0.000**	0.165	0.834
Blastp	0.462	0.537	**1.000**	**0.000**	0.183	0.816
Foldseek	0.898	0.101	**1.000**	**0.000**	0.845	0.154
Foldseek-TM	0.901	0.098	**1.000**	**0.000**	0.850	0.149
SS-predictor	0.950	0.049	**1.000**	**0.000**	0.924	0.075
PLMSearch	**0.989**	**0.010**	**1.000**	**0.000**	**0.983**	**0.016**

Supplementary Table 7 The recall rate of different methods for easy pairs and remote homology pairs. We selected the 5000 pairs with the highest similarity for different search methods and counted the recalled and missed pairs. As shown in Fig. 3 c-h in the main text, "Easy pairs" refers to the protein pairs with similar sequences and similar structures in the first quadrant. "Remote homology pairs" refers to the protein pairs with dissimilar sequences but similar structures in the fourth quadrant. "All pairs" refers to all protein pairs with TM-score > 0.5 in the first and fourth quadrants.

**General statistics**			
Dataset	Protein num	Cluster num	Pair num
SCOPe40-test	2,207	432	149,554
Swiss-Prot	430,140	6,086	3,852,993,796
**Big cluster statistics**			
Dataset	Protein num	Cluster num	Pair num
SCOPe40-test	305(13.8%)	1(**0.231%**)	92,720(**61.9%**)
Swiss-Prot	65,453(15.2%)	2(**0.032%**)	2,149,740,012(**55.7%**)
**Small cluster statistics**			
Dataset	2 proteins cluster num	1 protein cluster num (Singleton cluster num)	
SCOPe40-test	94	224	
Swiss-Prot	661	1,146	

Supplementary Table 8 Statistics of clustering results based on Pfam clan on SCOPe40-test and Swiss-Prot. The Big cluster in SCOPe40-test is CL0123. The Big clusters in Swiss-Prot are CL0023 and CL0063.

Methods	Input	Sensitivity	Speed	Query mode
		Sequence search		
MMseqs2	Sequence	Low	Very Fast	Multi query
Blastp	Sequence	Low	Very Fast	Multi query
HHblits	Profile HMMs	High	Slow	Single query
EAT	Per-protein embedding	Low	Very Fast	Multi query
pLM-BLAST	Per-residue embedding	Very High	Slow	Pairwise
		Structure search — structural alphabet		
Foldseek	Structure	High	Very Fast	Multi query
Foldseek-TM	Structure	Very High	Fast	Multi query
		Structure search — structural alignment		
TM-align	Structure	Very High	Slow	Pairwise
		Our methods		
SS-predictor	Per-protein embedding	High	Very Fast	Multi query
PLMSearch	Per-protein embedding	Very High	Very Fast	Multi query
PLMAlign	Per-residue embedding	Very High	Slow	Pairwise

Supplementary Table 9 Summary of the characteristics of search methods. According to the performance on the all-versus-all search test on SCOPe40-test, the methods are summarized according to their input, sensitivity, speed, and query mode.

	**Search methods**	**Alignment methods**
**Input**	Per-protein embeddings	Per-residue embeddings
**Speed**	Very fast	Slow
**Similarity**	Yes	Yes
**How to obtain similarity**	Fast retrieval based on similarity prediction between embeddings	Pairwise alignment based on SW/NW, obtaining similarity from alignment scores
**Query mode**	Multi query	Pairwise
**Alignment (global or local)**	No	Yes
**Representation method**	PLMSearch, EAT	PLMAlign, pLM-BLAST

Supplementary Table 10 Differences between search methods and alignment methods.

	Protein 1	Protein 2
**SCOPe ID**	d1oh0a_	d1ohpa_
**PfamFamily**	PF12680 (SnoaL_2)	PF02136 (NTF2)
**PfamClan**	CL0051 (NTF2)	CL0051 (NTF2)
**TM-score**	0.926	
**Structure**	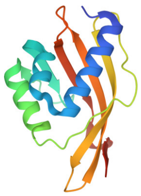	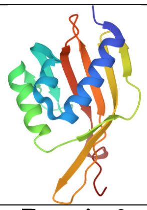
	**Protein 1**	**Protein 2**
**SCOPe ID**	d1ifca_	d2qo4a_
**PfamFamily**	PF00061 (Lipocalin)	PF14651 (Lipocalin_7)
**PfamClan**	CL0116 (Calycin)	CL0116 (Calycin)
**TM-score**	0.884	
**Structure**	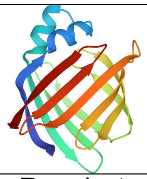	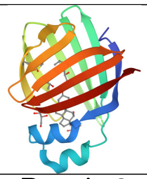
	**Protein 1**	**Protein 2**
**SCOPe ID**	d3c3ka_	d2gx6a_
**PfamFamily**	PF13377 (Peripla_BP_3)	PF13407 (Peripla_BP_4)
**PfamClan**	CL0144 (Periplas_BP)	CL0144 (Periplas_BP)
**TM-score**	0.880	
**Structure**	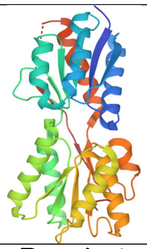	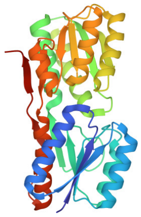
	**Protein 1**	**Protein 2**
**SCOPe ID**	d3k2aa_	d1akha_
**PfamFamily**	PF05920 (Homeobox_KN)	PF00046 (Homeodomain)
**PfamClan**	CL0123 (HTH)	CL0123 (HTH)
**TM-score**	0.876	
**Structure**	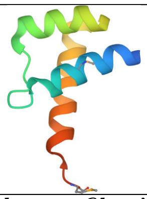	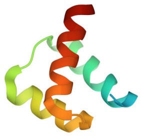

Supplementary Table 11 Case study for the pre-filtering results of PfamFamily & PfamClan. We investigated several protein pairs with TM-score>0.5 but missed by PfamFamily, and found that although the protein pairs do not share the domain belonging to the same family, the domain families belong to the same clan. Therefore, pre-filtering with PfamClan instead of Pfamfamily can help recall these protein pairs.

SS-predictor					
Similarity	0.1	**0.3**	0.5	0.7	0.9
Posterior probability(same fold)	0.000	**0.003**	0.456	1.000	1.000
Posterior probability(different folds)	1.000	**0.996**	0.543	0.000	0.000
COS					
COS	0.991	0.993	**0.995**	0.997	0.999
Posterior probability(same fold)	0.327	0.444	**0.717**	1.000	1.000
Posterior probability(different folds)	0.672	0.555	**0.282**	0.000	0.000
PLMAlign					
Score	3.0	5.0	7.0	9.0	**9.5**
Posterior probability(same fold)	0.001	0.020	0.285	0.545	**0.749**
Posterior probability(different folds)	0.998	0.979	0.714	0.454	**0.250**

Supplementary Table 12 Posterior probability of SS-predictor similarity, COS similarity, and PLMAlign score in SCOPe40-train. For SS-predictor, protein pairs with a similarity lower than 0.3 are usually assumed as randomly selected irrelevant protein pairs. For COS, the reference similarity of 0.995 is selected. For PLMAlign, the reference score of 9.5 is selected. See "Reference similarity" Supplement Section for more details.

	Query	Target	TM-score		Foldseek	SS-predictor
			Default	Avg. length	Probability	Similarity
UniProt ID	P32352	Q5HJR8	0.343	0.173	1.000	0.285
Length	222	745				
Structure	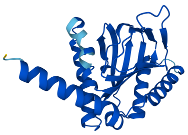	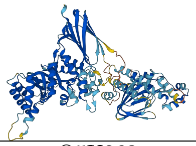				
UniProt ID	P32352	Q5U263	0.456	0.189	0.795	0.261
Length	222	1,146				
Structure	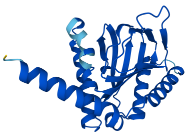	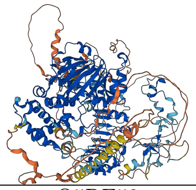				
UniProt ID	P32352	Q5RF50	0.375	0.190	0.975	0.268
Length	222	758				
Structure	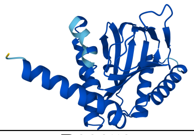	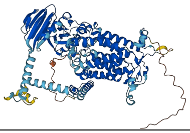				
UniProt ID	P32352	Q8NYT6	0.334	0.168	0.996	0.287
Length	222	745				
Structure	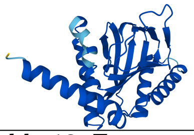	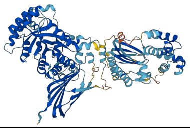				

Supplementary Table 13 Four protein pairs selected for the manual inspection. They are filtered by Foldseek but with a TM-score<0.2 (Wrong pairs, defined in Fig. 3b in the main text). TM-align(Default) uses the query protein length as the normalized length. TM-align(Avg. length) uses the average length of protein pairs as the normalized length. As reported in Foldseek's paper, Foldseek searches out these pairs because it focuses on local similarity. However, TM-align and PLMSearch focus on global similarity, so these pairs have TM-score<0.2 and similarity of SS-predictor lower than 0.3.

	Substitution matrix	Gap penalty	Scoring matrix	Traceback	Time
Smith-Waterman	Fix	Affine: 10+0.5*(L-1)	Truncate to zero	Begin with the highest score, end when 0 is encountered	-
Needleman-Wunsch	Fix	Affine: 10+0.5*(L-1)	Can be negative	Begin with the lower right of the matrix, end at top left	-
pLM-BLAST	Cosine	0	Can be negative	Traverse from all sequence boundaries	122,564 s
pLM-BLAST-global	Cosine	0	Can be negative	Begin with the lower right of the matrix, end at top left	18,812 s
PLMAlign	Dot Product	Linear: 1 * L	Truncate to zero	Begin with the highest score, end when 0 is encountered	12,796 s
PLMAlign-global	Dot Product	Linear: 1 * L	Can be negative	Begin with the lower right of the matrix, end at top left	12,470 s

Supplementary Table 14 Differences between the Smith-Waterman/Needleman-Wunsch algorithm, pLM-BLAST, and PLMAlign. The analysis was conducted in four steps: Substitution matrix, Gap penalty, Scoring matrix, and Traceback. The total alignment time spent for the all-versus-all search test on SCOPe40-test (4,870,849 pairs) is recorded. Smith-Waterman [1] and Needleman-Wunsch [2] algorithm take the implementation of EMBL-EBI (https://www.ebi.ac.uk) as an example.

Malisam	Number detected	F1	Recall	Precision
Sequence				
BLAST	2	0.000 ± 0.000	0.000 ± 0.000	0.000 ± 0.000
HMMER	3	0.000 ± 0.000	0.000 ± 0.000	0.000 ± 0.000
Needleman-Wunsch	129	0.032 ± 0.003	0.025 ± 0.002	0.043 ± 0.005
Smith-Waterman	129	0.033 ± 0.003	0.027 ± 0.002	0.044 ± 0.005
ProtT5-XL-UniRef50				
pLM-BLAST	129	0.039 ± 0.009	0.030 ± 0.007	0.065 ± 0.014
pLM-BLAST-global	129	0.169 ± 0.014	0.149 ± 0.012	**0.197 ± 0.016**
PLMAlign	129	0.154 ± 0.015	0.148 ± 0.014	0.161 ± 0.016
PLMAlign-global	129	**0.185 ± 0.014**	**0.178 ± 0.014**	0.194 ± 0.015
ESM-1b				
pLM-BLAST	129	0.013 ± 0.004	0.008 ± 0.002	0.035 ± 0.010
pLM-BLAST-global	129	0.047 ± 0.005	0.034 ± 0.004	0.082 ± 0.010
PLMAlign	129	0.048 ± 0.009	0.041 ± 0.008	0.060 ± 0.011
PLMAlign-global	129	0.129 ± 0.011	0.120 ± 0.010	0.142 ± 0.012

Malidup	Number detected	F1	Recall	Precision
Sequence				
BLAST	5	0.013 ± 0.013	0.006 ± 0.006	0.200 ± 0.200
HMMER	8	0.024 ± 0.024	0.013 ± 0.013	0.125 ± 0.125
Needleman-Wunsch	241	0.149 ± 0.011	0.121 ± 0.009	0.197 ± 0.014
Smith-Waterman	241	0.151 ± 0.011	0.123 ± 0.009	0.196 ± 0.014
ProtT5-XL-UniRef50				
pLM-BLAST	241	0.182 ± 0.017	0.157 ± 0.016	0.244 ± 0.020
pLM-BLAST-global	241	0.523 ± 0.018	0.479 ± 0.018	0.581 ± 0.019
PLMAlign	241	0.517 ± 0.020	0.499 ± 0.020	0.538 ± 0.021
PLMAlign-global	241	**0.560 ± 0.018**	**0.542 ± 0.017**	**0.582 ± 0.018**
ESM-1b				
pLM-BLAST	241	0.229 ± 0.019	0.222 ± 0.019	0.262 ± 0.021
pLM-BLAST-global	241	0.389 ± 0.021	0.343 ± 0.019	0.466 ± 0.022
PLMAlign	241	0.271 ± 0.021	0.258 ± 0.020	0.288 ± 0.021
PLMAlign-global	241	0.482 ± 0.018	0.464 ± 0.018	0.504 ± 0.018

Supplementary Table 15 Evaluation on remote homology alignment. F1, Recall, and Precision are counted based on whether the generated alignment and manual alignment are consistent at each position. The highest value achieved is highlighted in bold.

	**Protein**	**Protein pair**
**Training**		
**SCOPe40-train**	8,953	80,156,209 (8,953 * 8,953)<br>TM-score>0.5: 504,553<br>TM-score≤0.5: 79,651,656
**CATHS40**	21,474	28,440,312 (undersampled)<br>TM-score>0.5: 7,813,946<br>TM-score≤0.5: 20,625,460
**Test**		
**SCOPe40-test**	2,207	4,870,849 (2,207 * 2,207)
**New protein**	110	242,770 (110 * 2,207)
**Swiss-Prot**	430,140	43,014,000 (100 * 430,140)
**Target datasets on web server**		
**Swiss-Prot (unfiltered)**	568,744	query num * 568,744
**PDB**	679,875	query num * 679,875
**UniRef50**	53,625,855	query num * 53,625,855
**Evaluation on remote homology alignment**		
**Malisam**	233	129
**Malidup**	448	241

Supplementary Table 16 Datasets. By setting 0.4 sequence identity as the threshold to filter homologs, the max sequence identity of the test set relative to the training set does not exceed 0.4.

Methods	Similarity	Version
Sequence search		
MMseqs2	Bit score	Version 14.7e284
Blastp	Bit score	Version 2.12.0+
HHblits	Probability	Version 3.3.0
EAT	1 / (Embedding distance + 1)	Commit bcb935b
pLM-BLAST	Global similarity	Commit 0f226b0
Structure search — structural alphabet		
3D-BLAST-SW	E-value in ascending order	Beta102, with BLAST+ 2.2.26 and SSW version ad452e
CLE-SW	Score	PDB Tool v4.80, SSW commit ad452e
Foldseek	Probability	Version 6.29e2557
Foldseek-TM	Probability	Version 6.29e2557
Structure search — structural alignment		
CE	Z-score	BioJava's version 5.4.0
Dali	Dali's Z-score	DaliLite.v5
TM-align	TM-score	Version 20170708

Supplementary Table 17 Similarity and versions of baselines.

Methods	Source
	Sequence search
MMseqs2	https://github.com/soedinglab/MMseqs2
Blastp	https://anaconda.org/bioconda/blast
HHblits	https://github.com/soedinglab/hh-suite
EAT	https://github.com/Rostlab/EAT
pLM-BLAST	https://github.com/labstructbioinf/pLM-BLAST
	Structure search — structural alphabet
3D-BLAST-SW	http://3d-blast.life.nctu.edu.tw
CLE-SW	https://github.com/realbigws/PDB_Tool
Foldseek	https://github.com/steineggerlab/foldseek
Foldseek-TM	https://github.com/steineggerlab/foldseek
	Structure search — structural alignment
CE	https://github.com/biojava/biojava
Dali	http://ekhidna2.biocenter.helsinki.fi/dali
TM-align	https://seq2fun.dcmb.med.umich.edu/TM-align

Supplementary Table 18 Sources of baselines.

Methods	Family	Superfamily	Fold
	MMseqs2		
MMseqs2(Default)	0.157	0.021	0.000
MMseqs2(Best)	**0.318**	**0.050**	**0.002**
	Foldseek		
Foldseek(Default)	0.883	0.584	0.213
Foldseek(Best)	0.883	0.584	0.214
Foldseek-TM(Best)	**0.898**	**0.664**	**0.296**
	TM-align		
TM-align(Default)	0.859	0.529	0.158
TM-align(Avg. score)	0.933	0.711	0.326
TM-align(Avg. length)	**0.935**	**0.721**	**0.346**

Supplementary Table 19 Results with different settings for MMseqs2, Foldseek, and TM-align. Different settings can greatly affect sensitivity. MMseqs2(Default) and Foldseek(Default) are the default settings of the program. MMseqs2(Best), Foldseek(Best), and Foldseek-TM(Best) are the practiced parameters in the experiments of Foldseek [3]. TM-align(Default) uses the query protein length as the normalized length. TM-align(Avg. score) calculates TM-scores for both comparison directions and averages them together. TM-align(Avg. length) uses the average length of protein pairs as the normalized length. We experimented with the settings that yielded the highest sensitivity. The results and setting are consistent with the conclusions obtained from Foldseek [3] and MT-LSTM [4].

2 Supplementary Note

2.1 Sequence alignment

We define sequence identity the same as BLAST. Sequence identity could reflect the percentage of identical residues in the aligned sequence pairs. Sequence identity = (number of matched residues) / (the whole length of aligned sequences) [5]. We use the dynamic programming algorithm to perform pairwise sequence alignment and obtain the alignment with the highest sequence identity.

2.2 Reference similarity

Researchers often want to know what similarity approximately corresponds to the protein pairs sharing the same fold. Here, we address this issue by calculating the posterior probability for proteins at certain similarities sharing the same or different folds. We will examine the results of the posterior probabilities using the fold standards defined by SCOP. Protein pairs sharing the same fold are TPs. The experiments are performed with randomly selected 200 proteins from SCOPe40-train as queries and all proteins from SCOPe40-train as targets.

According to the Bayesian rules, for a given similarity, the posterior probabilities of proteins sharing the same or different folds can be expressed as:

$$\begin{cases} P(F \mid \mathrm{S}) = \frac{P(\mathrm{S}|F)P(F)}{P(\mathrm{S}|F)P(F)+P(\mathrm{S}|\bar{F})P(\bar{F})} \\ P(\bar{F} \mid \mathrm{S}) = \frac{P(\mathrm{S}|\bar{F})P(\bar{F})}{P(\mathrm{S}|F)P(F)+P(\mathrm{S}|\bar{F})P(\bar{F})} \end{cases} \tag{1}$$

Here, S stands for the similarity calculated by PLMSearch; F and $\bar{F}$ represent the events that the protein pair shares the same and different folds in SCOP, respectively; $P(F)$ and $P(\bar{F})$ are the prior probabilities. $P(\mathrm{S} \mid F)$ and $P(\mathrm{S} \mid \bar{F})$ are the conditional probabilities of similarity when the two proteins share the same or different folds, respectively. Thus, the conditional probabilities can be calculated by

$$\begin{cases} P(\mathrm{S} \mid F) = \frac{N(\mathrm{S})}{\sum N(\mathrm{S})} \\ P(\mathrm{S} \mid \bar{F}) = \frac{\bar{N}(\mathrm{S})}{\sum \bar{N}(\mathrm{S})} \end{cases} \tag{2}$$

where $N(S)$ is the number of protein pairs in the same fold with a certain similarity S, and $\bar{N}(S)$ is the number of protein pairs in the different folds with the similarity.

The prior probabilities $P(F)$ and $P(\bar{F})$ can be calculated by

$$\begin{cases} P(F) = \frac{N(F)}{N(F)+N(\bar{F})} \\ P(\bar{F}) = 1 - P(F) \end{cases} \tag{3}$$

where $N(F)$ and $N(\bar{F})$ are, respectively, the numbers of all the same and different folds pairs. Overall, $P(F) = 0.0104$ and $P(\bar{F}) = 0.9896$ in our counting.

The posterior probability for two proteins with a certain similarity to be in the same SCOP Fold is calculated by integrating the data of Equations 2 and 3 into Equation 1.

2.3 Remote homology alignment

2.3.1 PLMAlign pipeline

The procedure of PLMAlign, akin to the Smith-Waterman [1] and Needleman-Wunsch [2] algorithm, primarily encompasses the following three steps:

- Calculation of the substitution matrix — Use dot product to replace the original fixed substitution matrix.

  For a query protein of length m and a target protein of length n, the per-residue embeddings are $E_m(m * d)$ and $E_n(n * d)$ respectively. The corresponding substitution matrix $S_{mn}(m * n)$ is then obtained by the cross product of these two matrices.

$$S_{mn} = E_m \times E_n^T \tag{4}$$

  The essence of the cross product of the two matrices is that for the similarity $S_{mn}[i][j]$ between the i-th residue of the query protein and the j-th residue of the target protein, $S_{mn}[i][j]$ is calculated by the dot product of $E_m[i]$ and $E_n^T[j]$.

$$S_{mn}[i][j] = E_m[i] \cdot E_n^T[j] \tag{5}$$

  where $1 <= i <= m$ and $1 <= j <= n$. By replacing the original fixed substitution matrix with the similarity (dot product) between vectors, PLMAlign is able to capture the evolutionary information in

the context of residues and generates customized substitution matrices for each different query-target protein pair, resulting in more accurate alignments.

- Calculate the scoring matrix based on the substitution matrix and gap penalty — Linear gap penalty

  A linear gap penalty has the same scores for opening and extending a gap:

$$W_k = kW_1 \tag{6}$$

where W_1 is the cost of a single gap. The gap penalty is directly proportional to the gap length. When linear gap penalty is used, the Smith-Waterman algorithm can be simplified to:

$$H_{ij} = \max \begin{cases} H_{i-1,j-1} + s\left(a_i, b_j\right) \\ H_{i-1,j} - W_1 \\ H_{i,j-1} - W_1 \\ 0 \end{cases} \tag{7}$$

Compared with the traditional SW or NW algorithm using affine gap penalty (such as SW or NW implemented by EMBL-EBI (https://www.ebi.ac.uk)), the simplified algorithm uses $O(mn)$ steps, m and n are the lengths of the two sequences respectively. When an element is being scored, only the gap penalties from the elements that are directly adjacent to this element need to be considered, which greatly speeds up PLMAlign (Supplementary Table 14).

When performing a global comparison, the score can be negative, and the corresponding score matrix calculation formula is:

$$H_{ij} = \max \begin{cases} H_{i-1,j-1} + s\left(a_i, b_j\right) \\ H_{i-1,j} - W_1 \\ H_{i,j-1} - W_1 \end{cases} \tag{8}$$

- Search path based on scoring matrix — Same as traditional SW or NW algorithm.

  When performing a local comparison, PLMAlign begins with the highest score and ends when 0 is encountered. When performing a global comparison, PLMAlign begins with the cell at the lower right of the matrix and ends at the top left cell.

The differences between the SW/NW algorithm, pLM-BLAST, and PLMAlign are discussed in further detail in Supplementary Table 14.

2.3.2 Evaluation on remote homology alignment

Manual structure alignment is an intuitive human assessment, typically emphasizing 3D overlap, as these features are easier to visualize [6, 7]. All methods tend to concur when the sequence identity is high. As a result, the most valuable gold-standard alignment benchmark includes pairs with low sequence identity and varying degrees of structural similarity. Similar to DeepBLAST [8], our benchmarks were conducted on the curated Malisam [9] and Malidup [10] protein structural alignment benchmarking datasets, which are heavily skewed towards difficult-to-detect, low-sequence-identity remote homology pairs.

As depicted in Supplementary Fig. 6 and Supplementary Table 15, in both benchmarks, the majority of the protein pairs failed to pass the filtering steps of BLAST and HMMER. In other words, BLAST and HMMER were unable to detect the vast majority of the alignments. This left the Smith-Waterman [1] and Needleman–Wunsch [2] algorithm as the baselines. Owing to the use of dot products to calculate similarity instead of the original fixed substitution matrix, PLMAlign outperforms the Smith-Waterman and Needleman–Wunsch algorithm. Moreover, compared to pLM-BLAST, PLMAlign performs better on F1 and Recall, possibly because PLMAlign takes the gap penalty into account. Through time comparison (Supplementary Table 14), we discovered that PLMAlign is faster, particularly in local alignment. This may be primarily due to: (1) Dot product is faster than Cosine as no normalization is required. (2) PLMAlign uses a linear gap penalty model. When considering the gap penalty for a certain position, only the adjacent upper and left positions need to be considered (without considering the entire column and row). (3) For local alignment only, PLMAlign directly searches from the maximum value of the entire matrix, rather than searching in a traversal manner.

Additionally, we explored the impact of different language model embeddings (Supplementary Fig. 8). We compared the per-residue embeddings generated by ESM-1b and ProtT5-XL-UniRef50. We found that the COS distance between embeddings generated by ProtT5-XL-UniRef50 has better discrimination, both in self-alignment and alignment with another protein. Through experimental verification, we also found that ProtT5-XL-UniRef50 can yield better alignment results (Supplementary Table 15).

2.4 Baseline details

We first describe the similarity for sorting and versions of different methods in Supplementary Table 17, then summarize the sources of different methods in Supplementary Table 18.

2.4.1 Sequence search

- MMseqs2: A sequence search method with huge improvements in speed and sensitivity over other sequence search methods. For MMseqs2, different parameter settings will have a huge impact on the sensitivity. The default parameters (MMseqs2(Default)) lead to lower sensitivity (Supplementary Table 19). In order to ensure the fairness of the experiment, we used the parameters practiced in the Foldseek paper (–threads 56 -s 7.5 -e 10000 –max-seqs 2000) for experiments (MMseqs2(Best)).
- Blastp: We first downloaded Blastp from Anaconda: conda install -c bioconda blast. Then, we used the default parameters to build target datasets for SCOPe40-test and Swiss-Prot and searched against them. Taking the SCOPe40-test as an example, the command to build the dataset: makeblastdb -in protein.fasta -title scope40 -dbtype prot -out scope40 -parse_seqids. Search command: blastp -query protein.fasta -db scope40 -out search_result -outfmt "6 qacc sacc bitscore" -num_threads 56.
- HHblits: We first downloaded HHblits from Anaconda: conda install -c conda-forge -c bioconda hhsuite. Then, we used the default parameters to build target datasets for SCOPe40-test and searched against it. The steps are: 1. Download the UniRef30 database: wget https://gwdu111.gwdg.de/ compbiol/uniclust/2023_02/UniRef30_2023_02_hhsuite.tar.gz. 2. Build the SCOPe40-test dataset and search against it with HHblits according to the series of commands in "Building customized databases" from the wiki tutorial: https://github.com/soedinglab/hh-suite/wiki.
- EAT: We completed the following steps according to a series of commands in the repository: https://github.com/Rostlab/EAT. 1. Install 2. Use ProtT5-XL-U50 (or ProtT5 for short) to calculate the embedding of each residue ($L * 1024$ for ProtT5). The embeddings for each protein are derived by averaging the embeddings for each residue, resulting in a single 1024-d vector for each protein, regardless of its length, and the embeddings are stored as H5 files. 3. Calculate the inter-embedding Euclidean distance and sort according to 1 / (Embedding distance + 1) to complete the search.
- pLM-BLAST: We complete the following steps according to a series of commands in the repository: https://github.com/labstructbioinf/pLM-BLAST. 1. Install. 2. Use scripts/makeindex.py to generate index files from FASTA files. 3. Use the embeddings.py script to create the database. 4. Use dbtofile.py to create an additional file with flattened embeddings. 5. Use pLM-BLAST to search based on the generated embeddings.

2.4.2 Structure search — structural alphabet

- 3D-BLAST-SW: We utilized 3D-BLAST (beta102) with BLAST+ (2.2.26) and SSW [11] (version ad452e). We first transformed the PDB structures to a 3D-BLAST dataset using 3d-blast -sq_write and 3d-blast -sq_append. For Smith-Waterman, we employed (1) gap open of 8, (2) gap extend of 2, and (3) returning alignments (-c). (4) Use 3D-BLAST's optimized substitution matrix (-a 3DBLAST), and (5) Protein alignment mode (-p).
- CLE-SW: To convert the benchmark structure set to CLE sequences, we used PDB Tool v4.80 (github.com/realbigws/PDB_Tool). Following the conversion, we utilized SSW (commit ad452e) to align CLE sequences all-versus-all. We ranked the results based on the alignment score. The following parameters were used to execute SSW: (1) protein alignment mode (-p), (2) gap open penalty of 100 (-o 100), (3) gap extend penalty of 10 (-e 10), (4) CLE's optimized substitution matrix (-a cle.shen.mat), and (5) returning alignment (-c). DeepAlign [12] was used to infer gap open and extend values.
- Foldseek & Foldseek-TM: The latest protein structure search method, which achieves extremely high sensitivity in protein search by directly using structural information for encoding. Similarly, differences in parameter settings also affect the sensitivity of Foldseek (Supplementary Table 19). Again, we used the parameters practiced in the Foldseek paper (–threads 56 -s 9.5 -e 10 –max-seqs 2000) for experiments (Foldseek(Best)). Foldseek-TM then adds an additional parameter "–alignment-type 1".

2.4.3 Structure search — structural alignment

- CE: We utilized BioJava's [13] (version 5.4.0) implementation of the combinatorial extension (CE) alignment algorithm. We modified one of BioJava's modules in shape configuration to calculate the CE value. Our updated CEalign.jar file accepts a set of query files, the path to the target PDB files, and an output path as input parameters. This Java program executes an all-versus-all CE computation with an unrestricted gap size (maxGapSize -1) to improve alignment results [14].
- Dali: We installed DaliLite.v5. The input files for the SCOPe40 benchmark set were converted to DAT format. The conversion to DAT format resulted in 11,137 valid structures out of 11,211 initial structures for the SCOPe benchmark. After preparing the input files, we used Dali's structural alignment approach to calculate protein alignments.
- TM-align: We first downloaded TM-align from Anaconda: conda install -c bioconda tmalign. We ran the benchmark using "-a" parameters. So TM-align reports three TM-scores: (1) normalized by the

length of 1st chain (query), (2) normalized by the length of the 2nd chain (target), and (3) normalized by the average length of two structures. TM-align(Avg. length) uses the TM-score normalized by the average length of two structures and outperforms other settings (Supplementary Table 19). So the TM-score used in this paper is generated by TM-align(Avg. length).

Supplementary References

[1] Smith, T. & Waterman, M. Identification of common molecular subsequences. *Journal of Molecular Biology* **147** (1), 195–197 (1981) .

[2] Needleman, S. B. & Wunsch, C. D. A general method applicable to the search for similarities in the amino acid sequence of two proteins. *Journal of Molecular Biology* **48** (3), 443–453 (1970) .

[3] van Kempen, M. *et al.* Fast and accurate protein structure search with Foldseek. *Nat. Biotechnol.* (2023) .

[4] Bepler, T. & Berger, B. Learning the protein language: Evolution, structure, and function. *Cell Syst.* **12** (6), 654–669.e3 (2021) .

[5] Wang, Y., Wu, H. & Cai, Y. A benchmark study of sequence alignment methods for protein clustering. *BMC Bioinformatics* **19** (Suppl 19), 529 (2018) .

[6] Moult, J., Fidelis, K., Kryshtafovych, A., Schwede, T. & Tramontano, A. Critical assessment of methods of protein structure prediction (CASP)-Round XII. *Proteins* **86 Suppl 1**, 7–15 (2018) .

[7] Chothia, C., Novotný, J., Bruccoleri, R. & Karplus, M. Domain association in immunoglobulin molecules: The packing of variable domains. *Journal of Molecular Biology* **186** (3), 651–663 (1985) .

[8] Hamamsy, T. *et al.* Protein remote homology detection and structural alignment using deep learning. *Nat. Biotechnol.* (2023) .

[9] Cheng, H., Kim, B.-H. & Grishin, N. V. MALISAM: a database of structurally analogous motifs in proteins. *Nucleic Acids Research* **36**, D211–D217 (2007) .

[10] van Heel, A. J., de Jong, A., Montalbán-López, M., Kok, J. & Kuipers, O. P. BAGEL3: Automated identification of genes encoding bacteriocins and (non-)bactericidal posttranslationally modified peptides. *Nucleic Acids Res.* **41** (Web Server issue), W448–53 (2013) .

[11] Zhao, M., Lee, W.-P., Garrison, E. P. & Marth, G. T. SSW library: an SIMD Smith-Waterman C/C++ library for use in genomic applications. *PLoS One* **8** (12), e82138 (2013) .

[12] Jiménez-Moreno, A., Střelák, D., Filipovič, J., Carazo, J. & Sorzano, C. DeepAlign, a 3D alignment method based on regionalized deep learning for Cryo-EM. *Journal of Structural Biology* **213** (2), 107712 (2021) .

[13] Lafita, A. *et al.* BioJava 5: A community driven open-source bioinformatics library. *PLoS Comput. Biol.* **15** (2), e1006791 (2019) .

[14] Shindyalov, I. N. & Bourne, P. E. Protein structure alignment by incremental combinatorial extension (CE) of the optimal path. *Protein Eng.* **11** (9), 739–747 (1998) .
