# Supplementary material for: PLMSearch: Protein language model powers accurate and fast sequence search for remote homology
Source: Nat Commun. 2024 Mar 30;15:2775. doi: 10.1038/s41467-024-46808-5 (PMC10981738; doi:10.1038/s41467-024-46808-5)
Supplement: Supplementary file 3 — Reporting Summary [file 41467_2024_46808_MOESM3_ESM.pdf]

nature portfolio

Corresponding author(s): Jianyi Yang, Shanfeng Zhu

Last updated by author(s): Feb 9, 2024

Reporting Summary

Nature Portfolio wishes to improve the reproducibility of the work that we publish. This form provides structure for consistency and transparency in reporting. For further information on Nature Portfolio policies, see our Editorial Policies and the Editorial Policy Checklist.

Statistics

For all statistical analyses, confirm that the following items are present in the figure legend, table legend, main text, or Methods section.

n/a	Confirmed	
☐	☒	The exact sample size (*n*) for each experimental group/condition, given as a discrete number and unit of measurement
☐	☒	A statement on whether measurements were taken from distinct samples or whether the same sample was measured repeatedly
☒	☐	The statistical test(s) used AND whether they are one- or two-sided<br>*Only common tests should be described solely by name; describe more complex techniques in the Methods section.*
☒	☐	A description of all covariates tested
☒	☐	A description of any assumptions or corrections, such as tests of normality and adjustment for multiple comparisons
☐	☒	A full description of the statistical parameters including central tendency (e.g. means) or other basic estimates (e.g. regression coefficient) AND variation (e.g. standard deviation) or associated estimates of uncertainty (e.g. confidence intervals)
☒	☐	For null hypothesis testing, the test statistic (e.g. *F*, *t*, *r*) with confidence intervals, effect sizes, degrees of freedom and *P* value noted<br>*Give P values as exact values whenever suitable.*
☒	☐	For Bayesian analysis, information on the choice of priors and Markov chain Monte Carlo settings
☐	☒	For hierarchical and complex designs, identification of the appropriate level for tests and full reporting of outcomes
☐	☒	Estimates of effect sizes (e.g. Cohen's *d*, Pearson's *r*), indicating how they were calculated

Our web collection on statistics for biologists contains articles on many of the points above.

Software and code

Policy information about availability of computer code

Data collection	All of the protein search and alignment test were provided from the original benchmarks: Foldseek:https://www.nature.com/articles/s41587-023-01773-0, MALIDUP:https://pubmed.ncbi.nlm.nih.gov/17932926, MALISAM: https://pubmed.ncbi.nlm.nih.gov/17855399).<br><br>All baselines are open source methods. We download and implement them from their published links (including MMseqs2, Blastp, HHblits, EAT, pLM-BLAST, Foldseek, Foldseek-TM, TM-align, 3D-BLAST-SW, CLE-SW, CE, and Dali).<br><br>The versions used were MMseqs2 (Version 14.7e284), Blastp (Version 2.12.0+), HHblits (Version 3.3.0), EAT (Commit bcb935b), pLM-BLAST (Commit 0f226b0), Foldseek & Foldseek-TM (Version 6.29e2557), TM-align (Version 20170708), 3D-BLAST-SW (Beta102, with BLAST+ 2.2.26 and SSW version ad452e), CLE-SW (PDB Tool v4.80, SSW commit ad452e), CE (BioJava's version 5.4.0), and Dali (DaliLite.v5).<br><br>The weblinks used were:<br>MMseqs2: https://github.com/soedinglab/MMseqs2<br>Blastp: https://anaconda.org/bioconda/blast<br>HHblits: https://github.com/soedinglab/hh-suite<br>EAT: https://github.com/Rostlab/EAT<br>pLM-BLAST: https://github.com/labstructbioinf/pLM-BLAST<br>Foldseek & Foldseek-TM: https://github.com/steineggerlab/foldseek<br>TM-align: https://seq2fun.dcmb.med.umich.edu/TM-align<br>3D-BLAST-SW: http://3d-blast.life.nctu.edu.tw/<br>CLE-SW: https://github.com/realbigws/PDB_Tool

	CE: https://github.com/biojava/biojava<br>Dali: http://ekhidna2.biocenter.helsinki.fi/dali
Data analysis	PLMSearch is freely available at https://dmiip.sjtu.edu.cn/PLMSearch. PLMAlign is freely available at https://dmiip.sjtu.edu.cn/PLMAlign. PLMSearch and related tutorials are freely available to the public at GitHub https://github.com/maovshao/PLMSearch/blob/main/pipeline.ipynb. Reproducing our results and regenerating the main and supplementary figures requires only one file at GitHub https://github.com/maovshao/PLMSearch/blob/main/main.ipynb. The results of PLMSearch can also be reproduced through the capsule published on Code Ocean https://doi.org/10.24433/CO.8325548.v1. Run PLMAlign and reproduce the alignment experiment in Remote homology alignment Supplementary Section at GitHub https://github.com/maovshao/PLMAlign.<br><br>Structure visualizations were created in Pymol v.2.4.0 (https://github.com/schrodinger/pymol-open-source). For our PLMSearch data visualizations, we used Python version 3.8.16, with Seaborn Version 0.12.2, matplotlib-base Version 3.6.2.

For manuscripts utilizing custom algorithms or software that are central to the research but not yet described in published literature, software must be made available to editors and reviewers. We strongly encourage code deposition in a community repository (e.g. GitHub). See the Nature Portfolio guidelines for submitting code & software for further information.

Data

Policy information about availability of data

All manuscripts must include a data availability statement. This statement should provide the following information, where applicable:

- Accession codes, unique identifiers, or web links for publicly available datasets
- A description of any restrictions on data availability
- For clinical datasets or third party data, please ensure that the statement adheres to our policy

All of the protein sequences and structures used in this study for training and evaluation are publicly available (including SCOPe40, Swiss-Prot, AlphaFold Protein Structure Database, CATH, Malidup, Malisam, Pfam, ESM-1b, ProtT5-XL-UniRef50, PDB, and UniRef50).

The weblinks used were:
SCOPe40: https://scop.berkeley.edu
Swiss-Prot and UniRef50: https://www.uniprot.org
AlphaFold Protein Structure Database: https://alphafold.ebi.ac.uk/download
CATH: http://www.cathdb.info
Malidup: http://prodata.swmed.edu/malidup
Malisam: http://prodata.swmed.edu/malisam
Pfam: https://pfam.xfam.org, https://ftp.ebi.ac.uk/pub/databases/Pfam
ESM-1b: https://github.com/facebookresearch/esm
ProtT5-XL-UniRef50: https://github.com/agemagician/ProtTrans
PDB: https://www.rcsb.org

Training and test datasets used for PLMSearch can be found in https://dmiip.sjtu.edu.cn/PLMSearch/static/download/plmsearch_data.tar.gz. The data of PLMSearch can also be obtained and visualized through the capsule published on Code Ocean https://doi.org/10.24433/CO.8325548.v1. Training and test datasets used for PLMAlign can be found in https://dmiip.sjtu.edu.cn/PLMAlign/static/download/plmalign_data.tar.gz.

Research involving human participants, their data, or biological material

Policy information about studies with human participants or human data. See also policy information about sex, gender (identity/presentation), and sexual orientation and race, ethnicity and racism.

Reporting on sex and gender	The study does not involve any human participants or human data.
Reporting on race, ethnicity, or other socially relevant groupings	The study does not involve any human participants or human data.
Population characteristics	The study does not involve any human participants or human data.
Recruitment	The study does not involve any human participants or human data.
Ethics oversight	The study does not involve any human participants or human data.

Note that full information on the approval of the study protocol must also be provided in the manuscript.

Field-specific reporting

Please select the one below that is the best fit for your research. If you are not sure, read the appropriate sections before making your selection.

☒ Life sciences ☐ Behavioural & social sciences ☐ Ecological, evolutionary & environmental sciences

For a reference copy of the document with all sections, see nature.com/documents/nr-reporting-summary-flat.pdf

Life sciences study design

All studies must disclose on these points even when the disclosure is negative.

Sample size	SCOPe40-train & SCOPe40-test: Domains from SCOPe40 were split 8:2 by fold into SCOPe40-train and SCOPe40-test, and then domains with a single chain were reserved. We trained SS-predictor on SCOPe40-train and performed tests on SCOPe40-test as a benchmark. It is worth mentioning that each domain in SCOPe40-test belongs to a different fold from all domains in SCOPe40-train, so the difference between training and testing data is much larger than that of pure random division. We also studied the max sequence identity of each protein in SCOPe40-test relative to SCOPe40-train and found that the sequences in SCOPe40-test and SCOPe40-train are quite different, and most of the max sequence identity is between 0.2-0.3. The dataset used for training was all protein pairs in SCOPe40-train (8,953 proteins). TM-scores for all protein pairs between them were calculated. Therefore, a total of 80,156,209 query-target pairs were used to train SS-predictor. A all-versus-all search test was then implemented on the SCOPe40-test (a total of 4,870,849 query-target pairs were tested).<br><br>Swiss-Prot: A total of 542,317 proteins with both sequences and predicted structures were obtained. For these proteins, we dropped low-quality proteins with an avg. pLDDT lower than 70, and left 498,659 proteins. In addition, in order to avoid possible data leakage issues, like SCOPe40, we use 0.4 sequence identity as the threshold to filter homologs in Swiss-Prot from the training set. Specifically, we use the previously screened 498,659 proteins as query and SCOPe-train as the target dataset. We first pre-filtered potential homologous protein pairs using MMseqs2 and calculated the sequence identity between them. Among these protein pairs, as long as the sequence identity between the query protein in Swiss-Prot and any target protein is greater than or equal to 0.4, the query protein will be eliminated. Finally, a total of 68,519 proteins were eliminated in the homology screening, so a total of 430,140 proteins remained in Swiss-Prot used in our experiments. Subsequently, we randomly selected 50 proteins from Swiss-Prot and SCOPe40-test as query proteins (a total of 100) and searched for 430,140 proteins in Swiss-Prot as target proteins. Therefore, a total of 43,014,000 query-target pairs were tested.<br><br>CATHS40: We began with the CATHS40 non-redundant dataset of protein domains, which exhibits no more than 0.4 sequence similarity. Domains exceeding 300 residues were filtered out, leaving 27,270 domains. To prevent potential data leakage issues, akin to SCOPe40, we applied a 0.4 sequence identity threshold to filter homologs in CATHS40 from the testing dataset (SCOPe40-test and Swiss-Prot). Finally, 21,474 proteins in CATHS40 were left for training, and the max sequence identity of the test dataset to the new training dataset is still less than 0.4. We then undersampled CATHS40 domain pairs from different folds to acquire a substantial amount of training pairs with TM-scores above 0.5. Specifically, we sampled the TM-scores of 28,440,312 protein pairs for training, of which 7,813,946 pairs had a TM-score above 0.5.<br><br>Target datasets on web server: We currently have the following four target datasets for users to search: (1) Swiss-Port (568K proteins), the original dataset without filtering; (2) PDB (680K proteins); (3) UniRef50 (53.6M proteins). (4) Self (the query dataset itself).<br><br>Malisam and Malidup: Malidup encompasses 241 pairwise structure alignments, specifically targeting homologous domains within the same chain, thereby exemplifying structurally similar remote homologs. Malisam consists of analogous motifs.
Data exclusions	In SCOPe40, domains with multiple chains were excluded.<br>In Swiss-Prot, low-quality proteins with an avg.pLDDT of predicted structure lower than 70 and homologs from the training set with a sequence identity greater than or equal to 0.4 were excluded.<br>In CATHS40, Domains exceeding 300 residues and homologs from the training set with a sequence identity greater than or equal to 0.4 were excluded.
Replication	We performed our analyses on public datasets (including SCOPe, Swiss-Prot, AlphaFold Protein Structure Database, CATH, Malidup, Malisam, Pfam and ESM-1b). The analysis scripts are publicly available at https://github.com/maovshao/PLMSearch/blob/main/main.ipynb, https://github.com/maovshao/PLMAlign/blob/main/malidup.ipynb, https://github.com/maovshao/PLMAlign/blob/main/malisam.ipynb.
Randomization	Samples were allocated into different experimental groups randomly.
Blinding	Blinding is not relevant in this study, since there is no group allocation to assess a treatment effect.

Reporting for specific materials, systems and methods

We require information from authors about some types of materials, experimental systems and methods used in many studies. Here, indicate whether each material, system or method listed is relevant to your study. If you are not sure if a list item applies to your research, read the appropriate section before selecting a response.

Materials & experimental systems

n/a	Involved in the study	
☒	☐	Antibodies
☒	☐	Eukaryotic cell lines
☒	☐	Palaeontology and archaeology
☒	☐	Animals and other organisms
☒	☐	Clinical data
☒	☐	Dual use research of concern
☒	☐	Plants

Methods

n/a	Involved in the study	
☒	☐	ChIP-seq
☒	☐	Flow cytometry
☒	☐	MRI-based neuroimaging

Plants

Seed stocks	*Report on the source of all seed stocks or other plant material used. If applicable, state the seed stock centre and catalogue number. If plant specimens were collected from the field, describe the collection location, date and sampling procedures.*
Novel plant genotypes	*Describe the methods by which all novel plant genotypes were produced. This includes those generated by transgenic approaches, gene editing, chemical/radiation-based mutagenesis and hybridization. For transgenic lines, describe the transformation method, the number of independent lines analyzed and the generation upon which experiments were performed. For gene-edited lines, describe the editor used, the endogenous sequence targeted for editing, the targeting guide RNA sequence (if applicable) and how the editor was applied.*
Authentication	*Describe any authentication procedures for each seed stock used or novel genotype generated. Describe any experiments used to assess the effect of a mutation and, where applicable, how potential secondary effects (e.g. second site T-DNA insertions, mosiacism, off-target gene editing) were examined.*
